# A Comprehensive Review of Operative Considerations, Surgical Techniques, Outcomes, and Future Perspectives in Total Knee Arthroplasty

**DOI:** 10.7759/cureus.94345

**Published:** 2025-10-11

**Authors:** Abdulaziz Alzarooni, Rana Muhammad Ali, Aqsa Aftab, Faizan Vaja, Muhammad Abdulvahab, Vijayalakshimi Rajmohankumar, Obed Amoako-Adjei, Sher Bahadur Sunar, Gabriel G Hosu, Amena A Backosh, Bramaes Dahal, Fadi A Jamaleddin Ahmad

**Affiliations:** 1 Surgery, Tawam Hospital, Al Ain, ARE; 2 Orthopaedics, The Queen Elizabeth Hospital King's Lynn NHS Foundation Trust, King's Lynn, GBR; 3 Pulmonology, Holy Family Red Crescent Medical College and Hospital, Dhaka, BGD; 4 Orthopaedics, GMERS Medical College and Hospital, Junagadh, Junagadh, IND; 5 Orthopaedics, Salford Royal NHS Foundation Trust, Salford, GBR; 6 Medicine, Coventry University London, London, GBR; 7 Medicine, MAHSA University, Petaling Jaya, MYS; 8 General Surgery, Mid Yorkshire Teaching NHS Trust, Wakefield, GBR; 9 Internal Medicine, Worthing Hospital, University Hospitals Sussex NHS Foundation Trust, Worthing, GBR; 10 Orthopaedics, Dominica China Friendship Hospital, Roseau, DMA; 11 Orthopaedics, Medway Maritime Hospital, Gillingham, GBR; 12 Orthopaedics, East and North Hertfordshire Teaching NHS Trust, Stevenage, GBR; 13 Medicine, American University of the Caribbean School of Medicine, Cupecoy, SXM

**Keywords:** implant design, postoperative outcomes, preoperative optimization, robotic-assisted surgery, surgical techniques, total knee arthroplasty

## Abstract

Total knee arthroplasty (TKA) is still the gold-standard operative procedure for treating end-stage knee osteoarthritis, a disease impacting millions of patients worldwide and a key contributor to disability. As prevalence rates rise, the number of TKA procedures continues to increase. This review covers surgical procedure history, implant choices, perioperative guidelines, and technologies shaping TKA's future.

We performed a comprehensive narrative review of published literature on several aspects of TKA. Significant areas of comparison include cemented versus cementless fixation, mobile versus fixed bearing, posterior-stabilized versus ultra-congruent design, and single-radius versus multi-radius femoral design. The review encompasses preoperative optimization, alignment methods, robotic and sensor-aided surgery, postoperative rehabilitation, and outcomes. The data were collected from peer-reviewed journals, randomized controlled trials, clinical trials, and systematic reviews.

Cemented TKA remains the norm, but a cementless approach has equivalent outcomes in younger patients. Subtle functional benefits may exist with mobile-bearing designs, and newer designs, such as medial pivot implants, aim at optimizing kinematics. Robotic and sensor systems enhance implant accuracy and soft-tissue balance, providing short-term benefits such as improved function, reduced errors, and decreased blood loss. However, long-term outcomes, including implant durability and sustained function, remain uncertain. Optimizing the preoperative state and providing patient education have a significant impact on the postoperative state, and an early functional advantage is often associated with minimally invasive approaches. Several upcoming advancements, like AI-assisted surgery, outpatient arthroplasty, and 3D patient-specific printed implants, could positively influence the planning and execution of TKA. Nonetheless, their broad adoption is hindered by high costs, limited accessibility, and the requirement for additional validation via extensive, long-term studies.

These evidence gaps need to be filled before such technologies can be seamlessly incorporated into standard clinical routines. However, the aforementioned technologies and approaches require further studies to confirm their efficacy and safety. While TKA outcomes remain excellent, the continuous improvement of techniques, devices, and patient selection remains crucial to achieving optimal long-term outcomes with fewer complications.

## Introduction and background

Osteoarthritis (OA) was identified as the world's fourth leading cause of disability in 2020, impacting around 528-595 million people globally. It also ranks as the third most frequently diagnosed condition among older adults in primary care settings. Its prevalence is steadily increasing alongside global demographic aging, highlighting the growing public health challenge of OA in both developed and developing countries [1,2]. Knee OA is estimated to affect 3.48% of the population, with one-fourth of the affected individuals experiencing lifetime disability [1]. OA in the knee joint can lead to joint pain, loss of joint function, and poor quality of life. If damage to the joint and pain caused by OA are severe, then joint replacement surgery may be required. As the number of cases of knee OA continues to increase, the number of total knee arthroplasties (TKAs) will grow exponentially in the future [2,3]. There are various treatment options for knee OA, including non-pharmacological approaches such as exercise and lifestyle modifications, pharmacological treatments, analgesics, corticosteroid injections, and, as a last resort, surgical interventions [2].

The four surgical options for knee OA are osteotomy, unicompartmental knee arthroplasty (UKA), TKA, and arthroscopy. UKA, a joint resurfacing technique, was introduced in the 1970s. A 2018 study compared the outcomes of these four procedures, finding that osteotomy and UKA showed better functional improvements at six months and one year post-surgery, highlighting the advantages of osteotomy. UKA offers better short-term results, while TKA shows superior function scores after five years, indicating better long-term efficacy. TKA also has a high prosthesis survival rate and lower revision rate (3.7% at five years) compared to UKA (8%). Overall, TKA effectively relieves pain and restores joint function, with over 90% of patients experiencing satisfactory outcomes [2,4,5]. Soft tissue balance and mechanical alignment are crucial factors for the success of TKA; implant survival and knee function are among the key influencing factors [4]. Cemented TKA has been the accepted benchmark with a success rate of more than 90% [5,6].

With advancements in new designs, materials, and fixation methods, many studies suggest that cementless TKA offers outcomes that are comparable or even better than cemented TKA, especially in younger, high-demand patients with good bone quality who are suitable for biological fixation [1,6]. Additionally, comparative research has shown no significant differences between cemented and hybrid TKA concerning survival rates, complications, clinical scores, or radiological signs of loosening [7]. However, mobile-bearing TKA has advantages over fixed-bearing TKA in terms of Knee Society Knee Scores (KS-KS) and revision rates, and is generally more reliable and durable [3,8,9]. However, one study found no difference in clinical outcomes between the two groups [10].

With advancements in technology, newer techniques such as computer-navigated and robotic-assisted TKA have been developed to improve clinical and radiological outcomes [4]. However, there is no difference between computer-navigated and conventional TKA concerning clinical outcomes, scores, implantation accuracy, and long-term survival [11-13]. However, one study showed fewer postoperative leg alignment outliers and radiolucent lines compared to traditional TKA [4].

Our study aims to deliver a comprehensive review of recent developments in TKA, highlighting prospects. It uniquely combines comparisons of fixation methods, bearing designs, and alignment techniques within one framework to showcase innovations shaping the future of knee arthroplasty. Despite extensive research and widespread use, several key clinical uncertainties about the best approach to TKA remain. There is an ongoing debate over implant design choices, alignment methods (mechanical versus kinematic), and how to manage perioperative complications - all of which impact long-term function and patient satisfaction. Additionally, recent studies question traditional views on rehabilitation protocols and the optimal timing for surgery in patients with comorbidities. This review seeks to analyze current research to resolve these debates systematically, highlight gaps in the evidence, and suggest future directions for clinical practice and research in TKA.

## Review

Methodology

**Figure 1:**
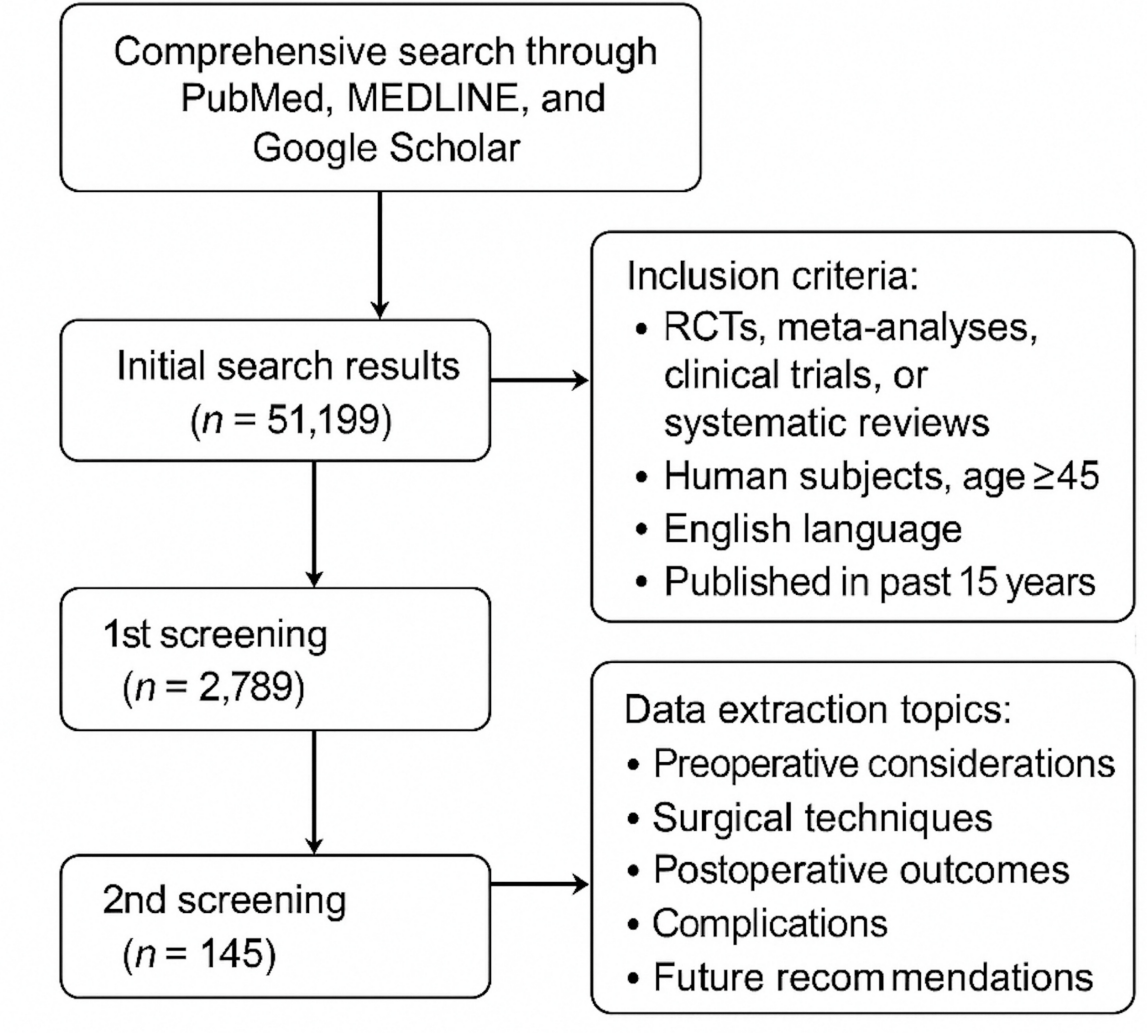
Screening of literature for the narrative review. RCT: randomized controlled trial

A comprehensive search was conducted through PubMed, MEDLINE, and Google Scholar to evaluate surgical techniques, outcomes, and future trends in TKA. The initial search produced 51,199 articles. In the first round of screening, studies were included if they were randomized controlled trials (RCTs), meta-analyses, clinical trials, or systematic reviews involving human subjects aged 45 and older and were published in English within the last 15 years. Studies using cadaveric and pediatric subjects, chemical studies, unicondylar or revision TKA operations, and articles not indexed in PubMed or non-English were excluded. This narrowed the list to 2,789 articles. The second round of screening refined the inclusion criteria to studies with clinical outcome scores, including the Knee Society Score (KSS), Western Ontario and McMaster Arthritis Index (WOMAC), Oxford Knee Score (OKS), or Knee Injury and Osteoarthritis outcome Score (KOOS), a minimum of 100 participants, a minimum five-year follow-up duration, and a focus on surgical techniques. Following this process, 145 articles were chosen for data extraction and included studies assessing preoperative considerations, surgical techniques, postoperative outcomes, complications, and future recommendations in TKA. The flowchart in Figure 1 highlights the process conducted in this review.

This narrative review was evaluated using the Scale for the Assessment of Narrative Review Articles (SANRA) to ensure methodological rigor and transparency. The SANRA scale assesses six domains - justification of the article’s importance, clarity of aims, description of the literature search, referencing, scientific reasoning, and data presentation - each scored from 0 (low) to 2 (high). Our manuscript received a perfect score of 12 out of 12 on the SANRA scale, demonstrating high quality across all evaluated areas: the article’s importance is justified by highlighting the increasing global burden of OA and the evolving role of TKA; aims are clearly stated in both the abstract and introduction, focusing on summarizing surgical innovations, evaluating clinical outcomes, and exploring future directions; the literature search methodology is explicitly described, including database selection (PubMed, MEDLINE, Google Scholar), eligibility criteria, screening process, and a Preferred Reporting Items for Systematic Reviews and Meta-Analyses (PRISMA)-style flowchart; referencing is thorough, current, and based on high-level evidence such as RCTs, meta-analyses, and registry data; scientific reasoning is logical and well-supported, involving multiple experts and authors to ensure scientifically credible work; and data presentation is straightforward, utilizing structured subheadings, summarized tables, figures, and flowcharts.

Preoperative considerations

To minimize perioperative complications and enhance the outcomes of TKA, patients need to undergo a thorough preoperative assessment, which helps identify modifiable risk factors, optimize comorbid conditions, and prepare the patient for surgery. Therefore, meticulous preoperative planning plays a crucial role, and the factors to be taken into consideration are as follows:

Patient Selection

Patient selection is the first critical step in preoperative considerations. It includes detailed history-taking, physical examinations, and an overall assessment of health status. Patients usually present with end-stage OA, post-traumatic osteoarthritis (PTOA), or symptoms refractory to conservative therapy [14].

Risk Stratification and Medical Optimization

Risk stratification and medical optimization are essential components of TKA's preoperative workup. Many patients undergoing this surgery are elderly and present with multiple comorbidities, making a thorough medical assessment necessary before surgery. There are several modifiable risk factors, namely obesity, malnutrition, diabetes, anemia, smoking, and opioid use, associated with adverse outcomes such as increased complications, infections, transfusion needs, etc. A systematic review, which was conducted, evaluated the outcomes of TKA in morbidly obese patients and found that this population is associated with higher risks, particularly for infections and revisions. In contrast, functional outcomes remain unchanged compared to the non-obese group [15]. A retrospective cohort study conducted for patients with type 2 diabetes mellitus suggests that preoperative HbA1c ≥ 8% may increase the incidence of wound complications [16]. In an extensive matched cohort study, anemic patients exhibited higher rates of significant complications, 30-day mortality, and extended length of stay as compared to non-anemic patients [17]. Similarly, patients with PTOA have higher infection and revision rates [18].

Therefore, these factors can be optimized, with evidence suggesting the following thresholds before surgery: BMI <40 kg/m^2^, serum albumin ≥3.5 g/dL, HbA1c ≤7.5%, hemoglobin ≥12 g/dL (women), ≥13 g/dL (men), and smoking cessation and ≥50% reduction of opioid use by four weeks before surgery [19]. Preoperative optimization, led by a physician assistant, demonstrated significant reductions in length of stay and cost of care, along with a decrease in complications [20]. Hence, detailed optimization protocols must be investigated, as the checklist alone is insufficient for risk reduction [21]. Examples of strategies for optimizing risk assessment are summarized in Table 1.

**Table 1:** TKA risk assessment and optimization for preoperative considerations. Data compiled by the authors from reference [19].

Methods	Purpose
Medical history	Helps identify comorbidities
Physical examination	Predicts functional outcomes and complications
Laboratory tests and imaging	Screens for healing risk, chronic illness, and imaging help in assessing the extent of damage and surgical planning
Infection screening	Prevents periprosthetic joint infections (PJI)
Cardiopulmonary workup	For anesthesia and surgery safety
Medication review (e.g., anticoagulants, immunosuppressants, steroids)	To check for risk of infection and bleeding

Imaging and Surgical Planning Tools

Preoperative imaging and surgical planning are integral to TKA. Specific considerations should be taken into account before proceeding with the surgery, one of which is the alignment strategy. A study of patients with pre-existing varus deformities demonstrated the efficacy of this strategy, suggesting favorable outcomes with mild residual varus alignment postoperatively [22,23]. Another essential consideration is the type of implant being used, such as a cementless or cemented fixation, a single-radius (SR) or multi-radius (MR) posterior-stabilized prosthesis, etc. Cementless fixation has shown lower revision and infection rates in recent data, as evidenced by a comprehensive retrospective review [1]. Meanwhile, a 10-year cohort study shows less notable anterior knee pain in the SR group as compared to the MR group [23].

Apart from the already mentioned factors, preoperative functional scores, such as KSS, Lower Extremity Activity Score (LEAS), Short Form 36 Health Survey (SF-36), and Western Ontario and McMaster Universities Osteoarthritis Index (WOMAC), are also calculated, as they play a crucial role in predicting perioperative outcomes, personalizing care plans, and guiding patients' expectations [24-26]. Additionally, routine standard radiographs should be obtained for TKA templating, primarily standing anteroposterior (AP), lateral, and skyline radiographs, which can help surgeons plan bone cuts, implant size, and position at the time of surgery, as well as recognize any rotational deformities [22].

Rotational Alignment

Alignment strategies are of crucial importance in determining the outcomes after surgery. Traditionally, the gold standard has been neutral alignment (0°-3°); however, newer evidence increasingly favors the alternative of residual mild varus alignment (3°-6°). Residual mild varus alignment tends to correlate with enhanced patient satisfaction, which translates into significantly higher Forgotten Joint Scores, with differences of approximately 6 points. This measure highlights improved subjective joint function and reduced awareness of the artificial joint during daily activities, implying better patient-perceived normalcy in mild varus alignment. By comparison, and in particular, severe varus alignment (≥6°) has uniformly provided poorer outcomes, such as substantially lower KS-KS with a decrease of approximately 3 points and significantly lower Knee Society Function Scores (KS-FS) with a reduction of about 8 points, implying compromised postoperative functional performance and less potential for implant longevity [24].

The rotational accuracy of TKA prosthetic components has emerged as a critical determinant of overall outcomes. Rotational mismatches have been strongly linked to suboptimal performance, characterized by increased pain, reduced functional performance, and patient dissatisfaction. The significant correlations (Spearman’s ρ between 0.44 and 0.68) between externally rotated tibial and femoral components and better KSS attest to the critical importance of being able to perform precise surgery. Accurate external rotation positioning significantly improves operative function and the patient’s level of satisfaction, thereby preventing revision surgery that may arise from malrotation-induced complications, such as patellofemoral instability and premature implant wear [26].

Patient Education and Prehabilitation

While every preoperative consideration is vital, properly educating patients about the entire process is paramount. Patients should be informed about all aspects, including any potential risk factors, the type of implant being used, and the chosen alignment strategy, along with the rationale behind these decisions. They should be well informed if there’s any possibility of complications regarding their specific condition, for example, obesity, diabetes, anemia, PTOA, etc., and should be counseled accordingly. Prehabilitation has been shown to enhance postoperative outcomes, with systematic reviews demonstrating improvements in knee flexibility, pain, and stiffness following surgery. However, the results across various studies are mixed, as some meta-analyses indicate only limited long-term functional advantages. Despite this variability, there is consistent evidence that prehabilitation leads to less postoperative pain, shorter hospital stays, and fewer inpatient rehabilitation admissions for patients participating in structured prehabilitation programs [25]. Now, the question is: What interventions can we take to achieve this goal? A structured prehabilitation program should be considered. Some steps that can be taken include physical training, which involves strengthening exercises (especially for the quadriceps and hamstrings), range-of-motion exercises, and balance and gait training. A nutrition plan to improve healing potential is another step that one can consider. Additionally, relaxation techniques or counseling sessions can help encourage a positive mindset among patients, thus reducing anxiety or any fear regarding surgery. On the other hand, a study shows no significant postoperative benefit of prehabilitation in function, pain, and quality of life in patients who underwent TKA; however, evidence suggests that prehabilitation may reduce admission to rehabilitation in this population [27].

Surgical techniques

TKA continues to evolve with surgical approaches, implant designs, and emerging technologies. Multiple factors affect the outcome of TKAs and patient satisfaction rates. Out of all, the alignment strategy remains the most debated topic, with debates centered on whether to follow a mechanical alignment, the standard technique, or the latest widely discussed kinematic or restrictive kinematic alignment. In recent years, newer functional alignments have also entered the debate. While mechanical alignment remains the most favored approach among surgeons, some patients with severe varus deformity preoperatively show good results with mild residual varus deformity. Longer comparative data are still awaited to show the superiority of either of the alignment strategies. Computer-assisted navigation and robotic assistance aim to improve implant placement precision and alignment, and they have been quite effective in achieving these goals. Nonetheless, multiple level I RCTs and several meta-analyses show that, although radiographic accuracy improves, there are no significant differences in clinical or functional results compared to traditional TKA [4,11-13].

Comparing the various TKA methods provides essential insights into patient outcomes, implant durability, and patient satisfaction. With TKA constantly evolving, the subtle differences among options become crucial for clinicians to make informed, patient-driven decisions.

Posterior Cruciate Ligament (PCL) Sacrificing TKA

Traditionally, the post-and-cam style posterior stabilized (PS) device has been used widely in PCL-sacrificing TKA; however, there have been recent developments of alternative options as well, such as congruent condylar-stabilizing (CS) and deep-dished ultra-congruent (UC) devices. There is no significant difference in clinical outcomes between the two devices; however, a trend favors the use of the UC device between six months and two years of follow-up [28].

SR and MR TKA

The MR femoral prosthesis was first developed in 1980, whereas the SR femoral prosthesis was developed in 1993. Both MR and SR yield good outcomes, but SR is associated with less knee pain compared to MR [23].

Infrapatellar Fat Pad (IPFP) Resection During TKA

The IPFP contains transverse infrapatellar arteries, and resecting the IPFP can lead to avascular necrosis and fracture of the patella. It also acts as a cushion between the anterior tibial plateau and the patellar tendon. Removing the IPFP can lead to complications, including anterior impingement, anterior knee pain, and reduced flexion after TKA [29].

Cemented Versus Cementless Fixation

**Figure 2:**
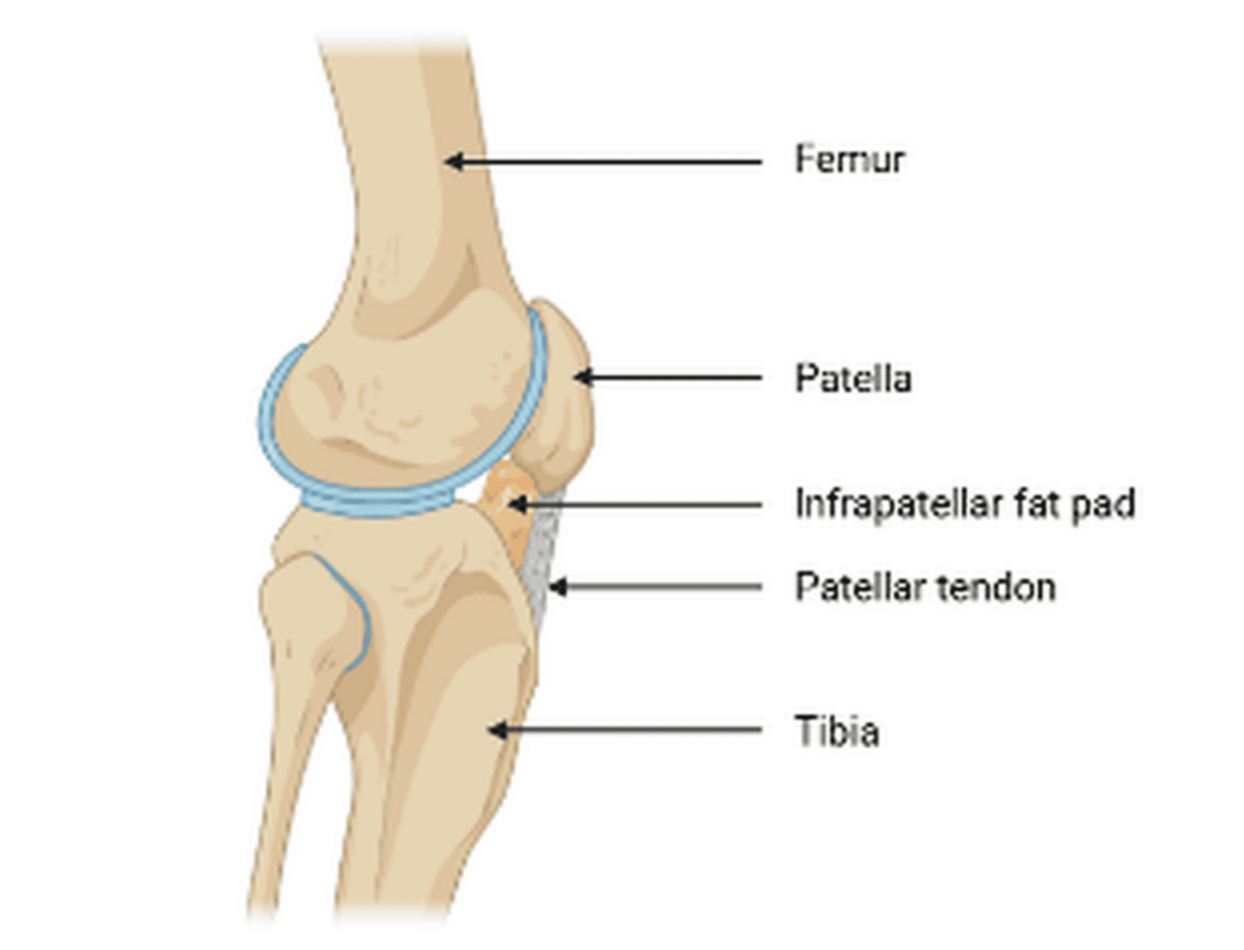
Lateral view of the knee joint illustrating the anatomical relationship among the femur, tibia, and patella. This image provides a visual reference for understanding alignment and joint mechanics relevant to total knee arthroplasty (TKA). Image credit: Created by the authors using BioRender (scientific illustration software).

**Figure 3:**
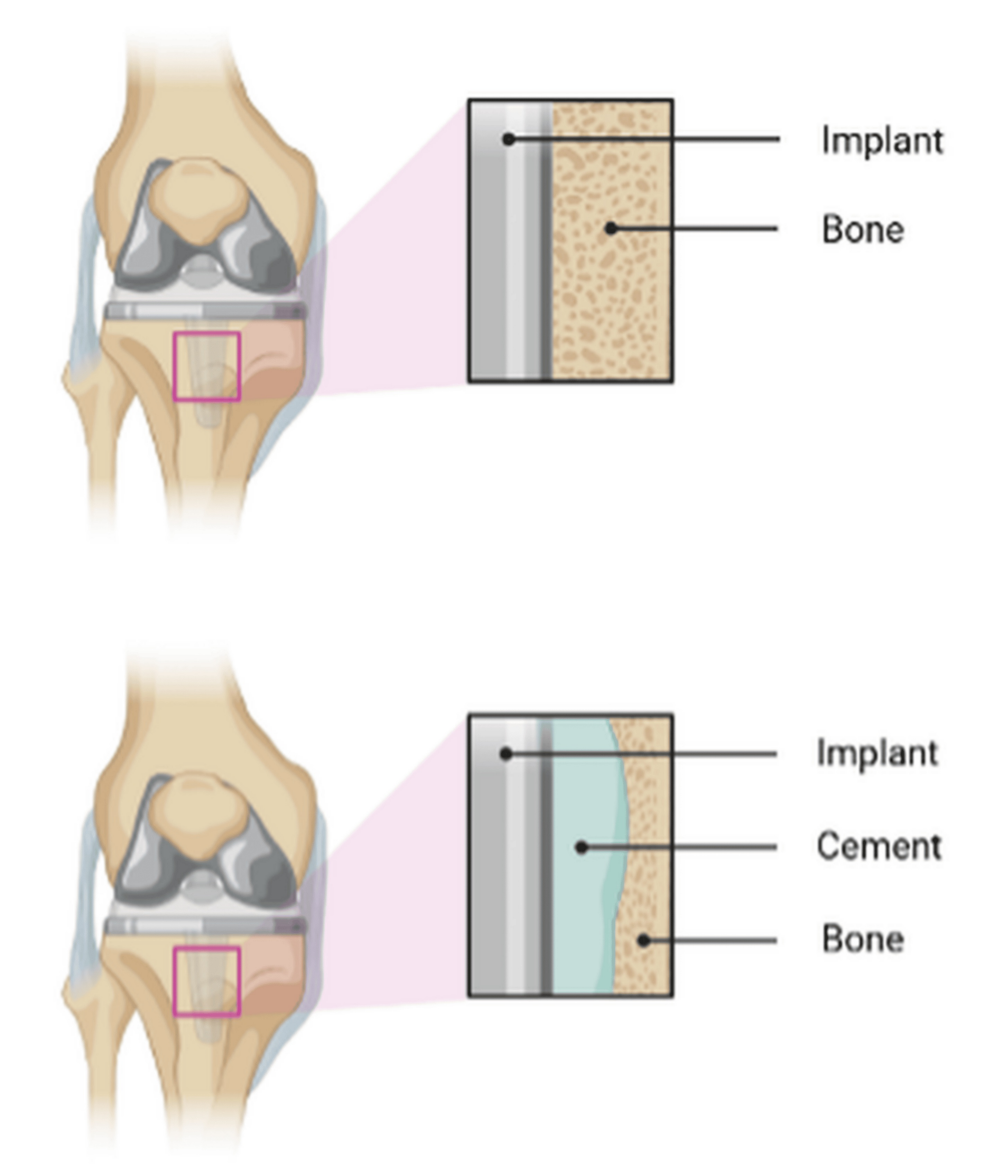
Schematic comparison of the implant-bone interface in cemented versus cementless total knee arthroplasty (TKA). The figure highlights key differences in fixation technique, showing the cement mantle in cemented implants and direct osseointegration in cementless designs, and emphasizes the potential challenges of cement removal during revision surgery. Image credit: Created by the authors using BioRender (scientific illustration software).

The debate surrounding cemented and cementless fixation in TKA continues. Cementless TKA has demonstrated better radiological performance, with significantly fewer radiolucent lines and considerably lower pain levels compared to cemented fixation, especially among younger patient groups. Research has shown a significant improvement in radiographic stability, indicating better bone-implant integration with the cementless technique, which is particularly valuable for younger, active patients. Nevertheless, no significant differences were found between cemented and cementless fixation when comparing functional outcomes using the KSS, range of motion, and patient self-reported measures. There was also no significant difference in the rate of aseptic loosening, indicating equivalent implant life for both fixation options, as observed in medium- and long-term follow-ups. Cemented fixation remains popular for immediate postoperative stability; however, the complexity of cement removal during revision procedures reflects significant difficulties, especially in patients with poor bone quality or those who are elderly [30]. Figure 2 shows a lateral view of the knee, and Figure 3 compares the cemented versus uncemented approach, highlighting the difficulties that can arise with radiological methods. This suggests that patients requiring multiple knee revisions may benefit from a cementless approach, which facilitates easier surgical revision and imaging.

Fixed-Bearing Versus Mobile-Bearing Prostheses

A comparison of fixed-bearing and mobile-bearing prostheses after approximately a decade of follow-up provides greater insight into implant longevity and functional outcomes. Mobile-bearing designs have had evident benefits in KSFS, most noticeably when PCL retention methods are employed. This improvement in functional scores, typically by about 5 to 7 points, reflects the potential for enhanced postoperative functionality and improved patient satisfaction with activities such as knee flexion, including stair climbing and squatting. Functional benefits of this kind notwithstanding, however, the rates of revision surgery, infections, and instability remain the same for mobile-bearing prostheses and fixed-bearing prostheses. Therefore, although functionally beneficial, mobile-bearing prostheses alone do not necessarily provide greater implant longevity or lower complication profiles than fixed-bearing designs [3].

Medial Pivot TKA

Recently, medial pivot TKA design has been gaining popularity among surgeons in developed countries. It mimics native knee kinematics, featuring a ball-and-socket mechanism on the medial side and gliding on the lateral side, similar to the native knee. Higher functionality and lower anterior knee pain have been reported. Multicenter studies and longer follow-ups are yet to be published. Another critical factor in implants is biomaterial science. As in total hip arthroplasty (THA), highly cross-linked polyethylene has gained superiority. In TKAs, polyethylene materials such as highly cross-linked polyethylene (HXLPE), ultra-high-molecular-weight polyethylene (UHMWPE), and vitamin E-infused polyethylene are being widely discussed. Studies have shown that regardless of material, bone resection and polyethylene thickness play a vital role in long-term implant survival and joint line preservation [1].

Robotic-Assisted TKA

Robotic-assisted TKA has introduced notable advancements in surgical precision, particularly in mechanical alignment and implant positioning. Compared to the conventional technique, robotic-assisted TKA is associated with significantly better functional outcomes, including improved WOMAC scores and a notable reduction in postoperative drainage by an average of 293 milliliters. Furthermore, robotic systems demonstrate greater accuracy in achieving optimal implant positioning and alignment, which is predicted to positively influence long-term implant survival and reduce the incidence of mechanical outliers [31]. In cruciate-retaining prostheses specifically, robotic assistance led to fewer radiological alignment errors and radiolucent lines on long-term follow-up, indicating superior radiological outcomes [4]. Compared to conventional techniques, robotic-assisted TKA has not demonstrated significant differences in operative time, complication rates, functional outcomes, implant survival, or aseptic loosening [11,13]. However, there is a slight improvement in the proportion of knees with ±3° deviation from a neutral mechanical axis in robotic TKA [13]. Both robotic and conventional groups report high levels of patient satisfaction and similar functional improvements, suggesting that increased surgical precision does not necessarily translate into superior clinical outcomes [4]. Thus, the optimal TKA strategy must be tailored, combining patient-specific priorities, precise surgical techniques, and advanced technology with evidence-based decision-making to achieve optimal postoperative quality of life and long-term implant success (Table 2).

**Table 2:** Comparison of surgical techniques. TKA: total knee arthroplasty; PCL: posterior cruciate ligament; KSFS: Knee Society Function Scores Data compiled by the authors from the following references: fixed vs. mobile bearing [8]; robotic-assisted TKA [13]; SR vs. MR [22]; IPFP [29]; cemented vs. cementless [30].

Technique	Key Features	Advantages	Notes
Single radius (SR) vs. multi-radius (MR)	SR is linked to less knee pain; both yield good functional results.	SR: Less anterior knee pain postoperatively.	Functional results are overall similar.
Infrapatellar fat pad (IPFP) resection	Resection can lead to patellar complications. Preservation is preferred for better pain outcomes.	Preserving IPFP reduces pain and complications.	Increased anterior pain and reduced flexion with resection.
Cemented vs. cementless fixation	Cementless yields better radiological results and lower pain, and similar functional scores and loosening rates to cemented.	Better bone-implant integration with cementless in young patients.	Cement removal during revision is difficult, similar to implant survival.
Fixed vs. mobile bearing	Mobile-bearing offers slightly better functional scores, especially with PCL retention; no difference in revision or complication rates.	Improved KSFS scores with mobile bearing.	No difference in complications or longevity.
Robotic-assisted TKA	Enhances implant positioning and alignment; improves radiological precision. No major difference in complications or satisfaction.	Better alignment, radiolucency, and accuracy.	Higher cost, no significant improvement in long-term outcomes.

Postoperative considerations

*Immediate Postoperative* *Considerations: Thromboprophylaxis and Physiotherapy*

Aspirin and enoxaparin are both frequently utilized after arthroplasty. A multicenter randomized trial found no significant difference in 90-day mortality rates - 1.67% for aspirin and 1.53% for enoxaparin. Nonetheless, thromboprophylaxis guidelines vary widely internationally. The American Academy of Orthopaedic Surgeons (AAOS) recommends aspirin as a safe and effective option for patients with a standard risk of venous thromboembolism (VTE). Conversely, the National Institute for Health and Care Excellence (NICE) recommends starting with low-molecular-weight heparin (LMWH) for 14 days, followed by a switch to aspirin for an additional 14 days after TKA. The Japanese Orthopaedic Association (JOA) guidelines suggest using LMWH or mechanical prophylaxis based on individual patient risk factors and bleeding considerations. These differences highlight the importance of tailoring prophylactic approaches to institutional protocols and regional guidelines, thereby maximizing safety and effectiveness.

Postoperative physical rehabilitation is crucial for achieving optimal recovery after TKA. Recent trials of structured physical activity intervention by physical therapists have proved effective. Wearable activity tracking, individualized daily step targets, and regular face-to-face feedback by physical therapists positively impacted patients’ moderate-to-vigorous physical activity (MVPA). Patients undergoing structured programs showed improvement during the six- and 12-month marks after hospital discharge [32].

Overall outcome and surgical complications

TKA is a surgical procedure performed primarily for severe OA in the knee. It is one of the most clinically successful and cost-effective surgical procedures developed in the last decade for end-stage OA [33]. A review of the current literature suggests that TKA leads to significant symptomatic relief for patients and improves their quality of life. This has been measured using patient-reported outcome measures (PROMs) such as the OKS, KSS, and Hospital for Special Surgery score (HSS), which have demonstrated impressive results among diverse populations and study cohorts [2,34]. A meta-analysis involving over 10,000 obese patients who underwent TKA revealed that patients reported sustained improvement in their function after a 4.8-year follow-up period. However, they had higher complication rates compared to non-obese patients [15].

Variability in outcomes has also been associated with surgical techniques (such as cemented vs. cementless), robotic techniques vs. conventional surgery, and implant design. A study demonstrating this is an RCT that compared UC and posterior-stabilized TKA designs, revealing better two-year outcomes with the UC implants [28]. Evaluation of the national registry of eight countries (USA, Germany, Australia, UK, Sweden, Norway, New Zealand, and the Netherlands) demonstrates that cemented TKA using antibiotic-loaded cement is associated with lower revision rates over 15-25 years compared to cementless implants [35]. Other RCTs have shown cementless TKA to have equivalent short-term outcomes but may have higher revision rates in the long term (over 10-17 years), especially among younger patients [36-38].

The most common and early complications of TKA, which typically appear within 90 days after surgery, include bleeding, wound infections, stiffness, thromboembolic disease, neural deficits, deep joint infections, ligament injuries, and instability. To improve clarity, these and subsequent postoperative complications are classified based on their timing and cause, as shown in Table 3 [33].

**Table 3:** Categorization of total knee arthroplasty (TKA) complications by timing (early vs. late) and underlying mechanism (biological vs. mechanical). DVT: deep vein thrombosis; PE: pulmonary embolism Data compiled by the authors from reference [33].

Category	Early Complications (<90 Days)	Late Complications (>90 Days)
Biological	Bleeding, superficial or deep wound infection, thromboembolic disease (DVT/PE), and/or neural deficit (e.g., peroneal neuropraxia)	Chronic deep joint infection, osteolysis, aseptic loosening, and/or periprosthetic fracture
Mechanical	Ligament injury, instability, and/or stiffness or arthrofibrosis	Polyethylene wear, patellar maltracking or fracture, component malalignment, and/or implant failure or breakage

One of the significant complications of concern post-TKA is infections, which have been shown to have a higher occurrence among patients with specific risks, such as obesity [15]. A large RCT of 2,893 knees found no reduction in infection rates with erythromycin- and colistin-loaded cement at a nine-year follow-up [39].

Patients with PTOA who undergo TKA are associated with increased complication rates and less satisfactory PROMs, as noted in a systematic review study [18]. In a study involving over 2,000 patients, cementless TKA was associated with higher rates of implant loosening and instability complications and higher revision rates [40].

Robotic-assisted TKA, a novel surgical technique, is shown to have the potential to improve efficiency and reduce alignment errors. A meta-analysis involving 1,942 knees reported improved functional scores and lower rates of complications in the short term compared to conventional surgical techniques [41]. However, no significant long-term advantage has been demonstrated, as evidenced by a study with a follow-up period of 13 years [13]. Patient-specific instrumentation (PSI) has also shown no superiority in terms of PROMs and revision rates compared to conventional techniques, as evidenced by an RCT spanning over 10 years [42]. Alignment is one of the primary differences in TKA surgical technique; a meta-analysis revealed no statistically significant difference in PROMs between neutral alignment and mild varus alignment [24].

Robotic-assisted techniques may have a beneficial impact on early recovery; however, they do not show a significant reduction in complication rates compared to conventional methods in the immediate and long term [41,43]. Additionally, mobile-bearing versus fixed-bearing TKA designs demonstrate comparable complication profiles at 10 years, with no discernible differences in revision rates [8]. Preoperative muscle strength is another individual-specific trait, aside from obesity, that influences outcomes. Studies have shown that preoperative exercise programs and postoperative rehabilitation can help mitigate complications and enhance functional recovery [38,44].

Future perspective

Numerous upcoming advancements are expected to impact TKA planning and execution positively. These innovations are categorized by their clinical development stage: established, emerging, and experimental, to highlight their current relevance and future potential.

Established Technologies

Minimally invasive and sensor-assisted TKA methods have shown measurable improvements in surgical accuracy and short-term results, such as better alignment, less blood loss, and quicker recovery, compared to traditional approaches. However, their long-term benefits remain uncertain, and high costs limit their broad use. More multicenter randomized trials with standardized outcomes are needed to verify their long-term clinical and economic benefits [41,45].

Emerging Technologies

Robotic-assisted surgery, AI-driven planning tools, and 3D-printed custom implants are at the cutting edge of TKA innovation. Robotic assistance has enhanced mechanical alignment, implant placement, and functional scores [41]. AI-based preoperative planning and 3D-printed implants also show promise in increasing surgical precision, customizing patient-specific kinematics, and reducing variability during surgery [46]. Despite these benefits, obstacles such as high costs, limited access in resource-poor settings, and the need for extensive validation through long-term, multicenter studies limit widespread adoption [41,46].

Experimental Innovations

Outpatient joint arthroplasty (OJA) and advanced AI predictive models are still in testing phases. OJA can reduce hospitalization costs and duration while maintaining patient satisfaction, but requires careful patient selection and institutional preparedness for safety and efficiency [45]. Similarly, next-generation AI algorithms designed to forecast surgical outcomes and improve perioperative workflows are primarily theoretical and require further clinical validation before being implemented on a large scale [46].

While established technologies already improve short-term results, emerging and experimental innovations hold great potential to transform TKA practice. Future research should focus on the cost-effectiveness, accessibility, and long-term validation of these technologies to ensure they lead to sustainable and equitable clinical benefits.

## Conclusions

The study shows that advances in technology have driven ongoing improvements in TKA surgical techniques, implant designs, and approaches. Evidence from multiple registry analyses indicates that uncemented TKA offers no significant benefit over cemented TKA in most cohorts; however, data from the USA and New Zealand joint registries reported lower revision rates with uncemented TKA. Similarly, meta-analytic evidence demonstrates no notable difference in outcomes between mobile-bearing and fixed-bearing TKA. Conversely, several comparative studies have reported that medial pivot TKA designs may enhance functionality and reduce the incidence of anterior knee pain; however, longer-term randomized data are still needed to confirm these advantages. The trend is shifting toward muscle-sparing subvastus and IFFP-preserving approaches, which is supported by short-term clinical studies showing faster rehabilitation, muscle strengthening, and reduced postoperative knee pain compared to traditional methods. Still, the medial parapatellar approach remains the preferred choice in obese patients. Despite its technical and radiological benefits, robotic-assisted TKA has not shown significant differences in operative time, complication rates, implant longevity, aseptic loosening, or long-term clinical results relative to conventional techniques. More multicenter research is required to assess the benefits of PCL preservation and the use of 3D-printed patient-specific implants and instruments in this procedure. Recent advancements, such as minimally invasive TKA and senior-assisted TKA, have demonstrated measurable improvements in surgical precision and short-term patient outcomes, supporting their role in clinical practice. In contrast, emerging technologies like AI and OJA remain largely investigational, with promising potential but limited clinical validation to date.

In summary, TKA has made significant progress in many areas; however, cutting-edge technologies remain less accessible due to higher costs and the need for specialized surgical skills. New implant designs show promise but have not yet proven superiority over traditional options. Future research, including higher-quality multicenter studies and extended follow-up periods, is essential.
